# Assessing the role of depression in reducing intimate partner violence perpetration among young men living in urban informal settlements using a mediation analysis of the Stepping Stones and Creating Futures intervention

**DOI:** 10.1080/16549716.2023.2188686

**Published:** 2023-03-17

**Authors:** Victoria Oyekunle, Andrew Gibbs, Andrew Tomita

**Affiliations:** a Department of Public Health Medicine, School of Nursing and Public Health, University of KwaZulu-Natal, Durban, South Africa; b Department of Psychology, University of Exeter, Exeter, UK; c Centre for Rural Health, School of Nursing and Public Health, University of KwaZulu-Natal, Durban, South Africa; d Gender and Health Research Unit, South African Medical Research Council, Pretoria, South Africa; e KwaZulu-Natal Research Innovation and Sequencing Platform (KRISP), College of Health Sciences, University of KwaZulu-Natal, Durban, South Africa

**Keywords:** Young men, depression, Stepping Stones and Creating Futures, intimate partner violence, informal settlement

## Abstract

**Background:**

Stepping Stones and Creating Futures (SS/CF) is a gender transformative and economic empowerment intervention that has effectively reduced the perpetration of intimate partner violence (IPV) by young men living in informal settlements in South Africa.

**Objective:**

This study examines whether depression mediated the association between SS/CF intervention and decreased IPV.

**Method:**

Data from a two-arm cluster randomised community-based controlled trial that evaluated the effectiveness of SS/CF in lowering IPV were obtained from 674 young men aged 18–30 within urban informal settlements in South Africa. After being randomly assigned to either the experimental arm (SS/CF) or the control arm, the participants were followed up for 24 months. Logistic regression using mediation analysis was conducted to see whether changes in depressive symptoms mediated the association between the intervention and reduced IPV perpetration.

**Results:**

Findings from the mediation analysis indicated that those assigned to the SS/CF experimental group reported lower depression (β = -0.42, *p* < 0.05) at 12 months, and this was subsequently associated with reduced IPV (β = 0.43, *p* < 0.05) at 24 months. The direct path from SS/CF to IPV was originally (β = -0.46, *p* < 0.01), but reduced in the mediation model to (β = -0.13, *p* = 0.50). Depressive symptoms mediated the association between the SS/CF intervention and decreased IPV perpetration.

**Conclusion:**

These findings suggest that one pathway through which SS/CF decreased IPV was through improvement in mental health (i.e. depression). Future IPV prevention interventions may consider incorporating components that focus on improving mental health as a way of also reducing IPV perpetration in disadvantaged settings.

## Introduction

There are over 800 million inhabitants of informal settlements globally [1]. The United Nations (UN) defines informal settlements as unplanned areas lacking basic infrastructure, with improvised shacks built on illegally subdivided land [2]. Despite significant progress in preventing their emergence, informal settlements continue to proliferate, and this issue plays a significant role in the global poverty crisis [2]. A large proportion of South Africa’s impoverished population live in informal settlements which are geographically spread across the nation, where over 1.2 million households live in such conditions without access to basic amenities [3].

People living in informal settlements are highly vulnerable to the overlapping issues of poor mental health and intimate partner violence (IPV), partly due to limited income and high rates of unemployment [4]. The prevalence of poor mental health is also particularly high in informal settlements and affects young men [5]. In a recent study conducted in informal settlements in Durban, South Africa, just under half (46.8%) of the young men had potentially clinically relevant symptoms of depression [5]. There are numerous pathways that may lead to high rates of poor mental health in these contexts, including exposure to violence in childhood and adulthood, high levels of substance misuse, as well as social marginalisation, which hinders young men from gaining respect and a sense of masculine achievement through providing for their families, which is a crucial component of masculinity in many societies [6,7]. Given the challenging social and economic circumstances in these settings, it is not surprising that IPV prevalence is high [6]. Young men may express their frustration and depression in a variety of ways, including by using violence against their intimate partners.

There is a strong relationship between men’s perpetration of IPV and depression, with studies reporting that men with depression are more likely to perpetrate IPV against their female partner, compared to men without depression [8–12]. A meta-analysis similarly showed that depression was an important predictor of IPV victimisation and perpetration [13]. Although the exact mechanisms through which depression influences IPV are unknown, it may be that depression is a manifestation of young men’s social marginalisation, and their sense of lack of ‘respect’ in their daily lives. It may follow that these young men try to gain respect through, for example, the control of women physically, sexually, and emotionally [14].

Given the link between poor mental health and IPV perpetration, there is an increasing focus on whether and how IPV prevention interventions targeting young men, may be mediated through mental health pathways. There is also limited knowledge regarding how potential interventions may improve the mental health of men in urban informal settlements in sub-Saharan Africa, in spite of the high incidence of IPV in these locations [15,16].

The combined Stepping Stones and Creating Futures (SS/CF) intervention was designed to address IPV perpetration in young adults aged 18–30 years living in urban informal settlements in South Africa [17]. The hypothesis was that SS/CF would reduce IPV perpetration through tackling inequitable gender norms and strengthening young men’s livelihoods. The intervention has already been evaluated in a cluster randomised controlled trial which showed reduction in IPV perpetration, strengthening of livelihoods, and reduction in alcohol use at 24 months [17]. In addition, qualitative research indicated that the reduction in IPV may be partially attributed to improved mental health [18]. Howeverto date, there has been no quantitative empirical evidence about whether mental health mediated reduction in IPV perpetration despite the high rate of depression in the target population. In this study, we test the hypothesis that the relationship between the SS/CF intervention and decreased IPV was mediated by improved mental health (as measured by depression status).

## Method

This analysis used data from the SS/CF intervention trial, a two-arm cluster randomised community-based controlled trial [17] conducted in urban informal settlements of Durban, KwaZulu-Natal (KZN) Province, South Africa between 2015 and 2019. This was done in collaboration with Project Empower (a local non-governmental organisation dedicated to addressing gender-based violence). Durban is the biggest city in KZN, the province being at the epicentre of South Africa’s HIV and IPV epidemics [19], with over three million residents [20] and 500 informal communities [21].

Trial eligibility was: male, aged 18–30 years, not in formal employment or education, living in selected informal settlement clusters, and being able to communicate in English, isiZulu, or isiXhosa. Those who lacked the ability to understand the information forms and were unable to provide written consent were therefore excluded.

Between September 2015 and September 2016, 674 consenting young men were recruited from 34 clusters. Before the intervention was implemented, study participants self-completed a questionnaire assessing their socio-demographic, mental health, economic status and IPV perpetration. All questionnaires were forward and back-translated between English and IsiZulu/IsiXhosa. Trained research staff who were fluent in English, isiZulu and isiXhosa were available to support questionnaire completion if needed. Of the 674 participants, 338 (50.2%) were randomly assigned to receive the intervention (SS/CF) and 336 (49.9%) to a wait-list control group. Data were collected at baseline, 12-month and 24 month post intervention. More information about the methodology, randomisation and study attrition can be found in the main trial protocol [22].

## Intervention

The SS/CF intervention has two components, the first component, Stepping Stones (SS), focuses on gender transformation, and consists of 10 three-hour sessions (total of 30 h) delivered to single-sex groups of 14–20 people over a five- to eight-week period. It specifically seeks to transform men’s perspectives and gender norms regarding how they interact with women, particularly with respect to IPV, enhancing gender equity, and relational and communication skills. The SS uses collaborative learning techniques, such as role plays and participatory drawing, and incorporates the participants’ daily lives into the exercises. Through these activities, participants start to think critically about how social contexts shape their behaviours and imagine alternative ways of relating to one another.

The second component, Creating Futures (CF), which focuses on economic empowerment, consists of eleven 3-h sessions (total of 33 h) delivered to the same single-sex groups as was the SS component. Establishing income and employment priorities, dealing with economic problems, making investments and savings, finding and sustaining jobs, and balancing work aspirations were among the topics discussed. The CF aims to assist young men in exploring the various resources they could use to establish and maintain a more secure means of survival.

## Measurement

### Outcome variable

The main outcome of the study was past year IPV perpetration, of which three types were identified: physical, sexual and emotional. IPV perpetration was assessed using South African adaptations from the WHO Multi-Country Study [23,24]. Physical IPV (5 items) focused on men’s perpetration of behaviourally specific actions, such as slapping, kicking, pushing or shoving, choking, burning or threatening their partner with weapons in the previous 12 months. Sexual IPV (three items) focused on perpetration relating to forced non-consensual sex, and using threats to get sex, in the previous 12 months. Emotional IPV (five items) focused on perpetration using verbal insults, humiliation in front of other people, yelling or smashing things, intimidation, threatening to hurt, and destroying things of importance to the partner in the previous 12 months. All items were based on a 4-point scale, where 1 = ‘never’; 2 = ‘once’; 3 = ‘few’; 4 = ‘many’. In the main analysis the three forms of IPV were merged as one binary IPV outcome variable, with any positive response (i.e. once, few, or many) to any IPV was coded as IPV perpetration (i.e. 0 = ‘no IPV’; 1 = ‘presence of IPV’). In additional analyses, the three forms of IPV were kept separate with similar binary coding. Internal consistency of the instrument was good (Cronbach alpha = 0.91).

### Mediating variable

Symptomatology of depression was assessed using the Centre for Epidemiological Studies-Depression (CES-D) 20-item scale [25]. Previously used in South African informal settlements [26], respondents were asked about their emotions and experiences within the past week. Possible responses were based on a 4-point Likert scale where 0 = ‘rarely or none of the time’; 1 = ‘some or a little of the time (1–2 days)’; 2 = ‘moderate amount of time (3–4 days)’; 3 = ‘most or all the time (5–7 days)’, with items summed. Total scores range from 0 to 60, with a cut-off point of 21 or larger denoting significant depressive symptoms (hereafter labelled as depression). The internal consistency of the instrument was good (Cronbach alpha = 0.87).

### Socio-demographic and clinical characteristics

Baseline socio-demographic characteristics consisted of age, employment, education and relationship status. Five clinical characteristics were food security, exposure to potentially traumatic events, participation in crime, adverse childhood events (ACE), and problematic alcohol use. The inclusion of these characteristics allowed us to assess the representativeness of the target population as they also have the potential to affect health outcomes.

#### Food security

The Household Hunger Scale (HHS), which has been used in informal settlements in South Africa [27], was included to assess the level of food security [28]. Three questions about difficulties with food insecurity over the previous month were asked, the possible responses ranging from: 0 = ‘never’; 1 = ‘rarely’; 2 = ‘sometimes’; and 3 = ‘often’; which were further recoded into three (never or rarely = ‘none or little’; sometimes = ‘moderate’; often = ‘high’), as recommended by Deitchler and colleagues [28]. Based on the Cronbach alpha, the instrument’s internal consistency was 0.83.

#### Exposure to potentially traumatic events

The adapted Life Event Checklist from the post-traumatic stress disorder checklist, which has been validated in other South African populations [29], assessed potentially traumatic exposures. The self-report instrument is an 8-item scale designed to screen for potentially traumatic experiences in a respondent’s life. Examples of items in the scale included ever witnessed a murder, ever being tortured, robbed, or kidnapped. Participants answered no = ‘0’ or yes = ‘1’ to all question; responses were summed up to 8, which was further recoded as 0 = ‘no exposure’ and 1–8 = ‘presence of exposure’. The internal consistency based on the Cronbach’s alpha is 0.72.

#### Participation in crime

The 10-item participation in crime scale, which has been previously used in informal settlements in South Africa [26], evaluated crime involvement and participation. It questioned men about their involvement in a variety of criminal behaviours over the previous 12 months. Based on a 3-point Likert like scale, responses included: 1 = ‘never’; 2 = ‘once’; 3 = ‘more than once’. Binary recoding was done, where people who answered ‘never’ to all questions were coded as (0 = ‘never having committed a crime’), while those who answered otherwise to one or more questions were coded as (1 = ‘crime perpetrators’). Based on the Cronbach alpha, the instrument’s internal consistency was 0.87.

#### Adverse childhood events (ACE)

The Childhood Trauma Questionnaire [30], a 13-item scale, was used to assess ACE, the instrument having been modified and adapted previously for use in South Africa [29]. The respondents were asked about potentially traumatic childhood events, their possible response being based on a 4-point Likert-like scale, where 1 = ‘never’; 2 = ‘sometimes’; 3 = ‘often’; 4 = ‘very often’. The responses were grouped and recoded into two categories: no = never experienced it, and yes = experienced it (responses 2–4). After recoding, scores ranged from 0 to 12, with 0 = ‘no adverse experiences’; 1–3 = ‘few adverse experiences’; and 4–12 = ‘many adverse experiences’. Based on the Cronbach alpha, the instrument’s internal consistency was 0.86.

#### Problematic alcohol use

The Alcohol Use Disorders Identification Test (AUDIT) scale [31] was used to assess harmful alcohol use, which has been previously used in South African informal settlements [24]. Respondents were asked 10 questions about their alcohol consumption in the previous year, with possible responses based on a 5-point Likert scale, with 1 = ‘never, 2 = ‘less than monthly, 3 = ‘monthly’, 4 = ‘weekly’, and 5 = ‘daily or almost daily’. Scores were summed (range 0–60) and divided into two categories, i.e. 0–7 = ‘no harmful alcohol use’ and 8–60 = ‘harmful alcohol use’. Based on Cronbach alpha, the internal consistency was 0.79.

## Data analysis

We examined whether depression symptoms (at 12-months) mediated the relationship between exposure to the intervention (baseline) and IPV outcome (at 24-months). Firstly, we present baseline descriptive statistics of the population sample, which included frequency and percentage distributions of the socio-demographic characteristics. Secondly, we conducted logistic regression, using binary mediation analysis (with binary outcome and binary mediator variable structure) to see whether changes in depressive symptoms mediated association between assignment to the intervention and IPV based on a three-step approach described by MacKinnon, Fairchild, and Fritz [32]. In the first mediation analysis step, we assessed the total effect (referred to as ‘path c’ by MacKinnon, Fairchild, and Fritz [32]) of assignment to SS/CF on IPV, but without the mediating variables, using logistic regression. In the second mediation analyses step, we then assessed the impact of assignment to SS/CF based on depressive symptoms at 12-months (referred to as ‘path a’) using logistic regression. In the final mediation step, we assessed the direct effect (referred to as ‘path c’) of the impact of assignment to SF/CF on IPV between the two groups, controlling for mediating variable, i.e. depression status (referred to as ‘path b’), using logistic regression. If association between SS/CF and IPV was mediated by changes in depression symptoms, we would expect to see a significant reduction in the effect of the intervention when the mediators were controlled for in the model (path c’), compared with the total effect without mediators (path c). In addition, we would also anticipate a significant association between depression status (path b) on IPV outcome. The total indirect effect of SS/CF was computed as the difference between the total effect (c) and the direct effect (c’). Furthermore, separate mediation analyses were undertaken for the three specific types of IPV (emotional, sexual and physical) to determine which IPV was best mediated by improvement in depression. All regression analyses controlled for socio-demographic variables. STATA 17 was used for all analyses.

## Results

Between September 2015 and September 2016, 674 young men were enrolled across 34 clusters (Table 1). At baseline, men between the ages of 20 and 24 made up half of the sample (n = 349, 51.8%), and nearly two-thirds had not worked in the past three months (n = 433, 64.3%). Education levels were low, 11.4% (n = 77) had only finished primary education and most reported only having secondary school, incomplete (n = 391, 58.0%), and 11.4% (n = 77) had only finished primary education. The majority (78.6%, n = 530) reported having a current intimate relationship.

**Table 1.** Socio-demographic and other baseline characteristics by study arm.

		Overall	Intervention	Control
		% (n=674)	% (n=338)	% (n=336)
Age group (years):	18–19	10.7 (72)	8.6 (29)	12.8 (43)
	20–24	51.8 (349)	53.9 (182)	49.7 (167)
	25–29	30.9 (208)	32.8 (111)	28.9 (97)
	30 and above	6.7 (45)	4.7 (16)	8.6 (29)
Employment Status:	Not worked in the past 3 months	64.3 (433)	62.1 (210)	66.6 (223)
	Worked in the past 3 months	35.7 (240)	37.9 (128)	33.4 (112)
Education Status:	Incomplete secondary	69.4 (468)	69.2 (234)	69.6 (234)
	Complete secondary	30.6 (206)	30.8 (104)	30.4 (102)
Relationship Status:	None	21.4 (144)	23.7 (80)	19.1 (64)
	In a relationship	78.6 (530)	76.3 (258)	81.0 (272)
Food Security:	Little/no hunger in the household	18.6 (125)	20.4 (69)	16.7 (56)
	Moderate hunger in the household	56.5 (380)	53.9 (182)	59.1 (198)
	Severe hunger in the household	25.0 (168)	25.7 (87)	24.2 (81)
Crime Status:	Never committed a crime	45.3 (305)	47.3 (160)	43.2 (145)
	Crime perpetrators	54.8 (369)	52.7 (178)	56.9 (191)
Adverse Childhood Event (ACE):	No trauma	7.7 (52)	7.4 (25)	8.0 (27)
	Mild trauma	32.5 (219)	33.4 (113)	31.6 (106)
	Severe trauma	59.8 (403)	59.2 (200)	60.4 (203)
Traumatic Exposure:	No exposure	78.6 (526)	78.8 (264)	78.4 (262)
	Presence of exposure	21.4 (143)	21.2 (71)	21.6 (72)
Gender Attitude (GEM):	Low equity	33.5 (225)	33.4 (113)	33.5 (112)
	Moderate equity	36.6 (246)	35.8 (121)	37.4 (125)
	High equity	29.9 (201)	30.8 (104)	29.0 (97)
Alcohol level (AUDIT):	Sensible drinking	56.4 (380)	58.6 (198)	54.2 (182)
	Alcohol problem	43.6 (294)	41.4 (140)	45.8 (154)
Depression:	No depression	53.2 (356)	54.3 (182)	52.1 (174)
	Presence of depression	46.8 (313)	45.7 (153)	47.9 (160)
IPV Perpetration	No IPV	49.4 (333)	55.3 (187)	43.5 (146)
	Presence of IPV	50.6 (341)	44.7 (151)	56.6 (190)

In terms of food security, 56.5% (n = 380) and 25.0% (n = 168) reported moderate and severe hunger, respectively. About a fifth of men reported any potential traumatic events (n = 143, 21.4%), while more than half had a history of crime perpetration (n = 369, 54.8%) and the majority reported multiple adverse childhood experiences (n = 403, 59.8%). Almost half reported harmful alcohol use (n = 294, 43.6%). Just under-half reported significant symptoms of depression (n = 313, 46.8%), and 50.6% (n = 341) reported perpetrating IPV in the past year.

**Figure 1.**
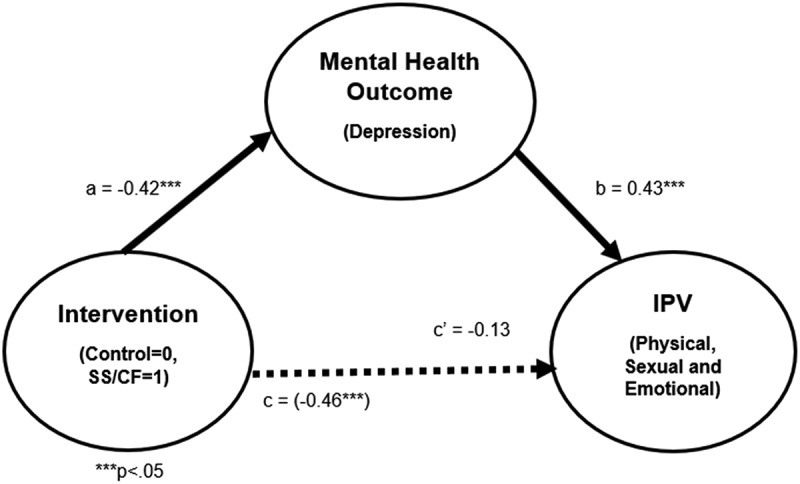
Effects of SS/CF and reduced depressive symptoms on IPV perpetration, controlling for socio-demographic variables.

The results (Figure 1) revealed that the total effect of SS/CF on IPV without the mediating variable (path c: adjusted β = −0.46, *p* < 0.01) indicated a significant reduction in the men’s perpetration of IPV. The impact of SS/CF on IPV became insignificant (path c’: adjusted β = −0.13, *p* = 0.50) with the inclusion of the depression status as a mediating variable, but we detected an indirect effect of SS/CF on IPV through depression. We found a relationship between assignment to SS/CF and depression (path a: adjusted β = −0.42, *p* < 0.05), indicating SS/CF reduced depressive symptoms at 12-months, as well as positive association between depression at 12-months and IPV at 24-months (path b: adjusted β = 0.43, *p* < 0.04). Given the significant association in path a, b and c (but not in path c’), this indicates that the relationship between SS/CF and reduced IPV perpetration was somewhat mediated by improved depression status at 12-months. When we separated out the different forms of IPV, this result of depression mediating the relationship only held true for the impact of SS/CF on emotional IPV (Table 2).

**Table 2.** Effects of SS/CF and reduced depressive symptoms on the different forms of IPV, controlling for socio-demographic variables.

IPV	Path	Coefficient	95% CI
Emotional	c	−0.43***	−0.74, −0.11
	a	−0.42***	−0.82, −0.01
	b	0.44***	0.03, 0.85
	c’	−0.11	−0.50, 0.28
Sexual	c	−0.39***	−0.77, −0.00
	a	−0.42***	−0.82, −0.01
	b	0.34	−0.15, 0.83
	c’	−0.2	−0.68, 0.27
Physical	c	−0.43***	−0.77, −0.09
	a	−0.42***	−0.82, −0.01
	b	0.25	−0.19, 0.70
	c’	−0.24	−0.67, 0.18

***p < 0.05

## Discussion

Although past research has shown that SS/CF is effective in reducing men’s perpetration of IPV [17], causal mechanisms had not previously been explored. In this investigation, we provided evidence that depression is one mediating relationship between the SS/CF intervention and IPV perpetration. The causes of IPV are complex and include poverty, gender inequitable masculinities, substance misuse and childhood adverse experiences [6]. Given that men with depression are more likely to perpetrate IPV against their female partners [13,33], our work suggests that it may be important for IPV prevention interventions to include components addressing depression, alongside broader gender-transformative and economic empowerment interventions.

There are several plausible explanations as to why depression may mediate the impact of SS/CF on IPV perpetration. First, gender inequitable masculinities impose a psychological burden on men [34], and it may be that aspects of these were addressed in the intervention, as SS/CF focused on supporting men to imagine alternative forms of masculinity. This may have led to reduced men’s psychological burden, potentially decreasing depression and lowering IPV. Second, the group-based nature of the intervention may have led to improved social connections between men, reducing depression. Studies have shown that men who have more conservative gender norms, are more socially isolated (to maintain a certain image of independence and strength) [35,36], and social isolation is associated with poor mental health including depression [37]. Although data are not available, it is also possible the intervention was instrumental in improving social connection as it created a supportive environment for discussion and skill development. A third potential pathway could have been that depression mediated the relationship because of the impact of SS/CF on improved livelihoods. Research shows a strong relationship between poverty and poor mental health [38–40]. SS/CF strengthened men’s livelihoods, and it could have been this which led to the reduction in depression, and then IPV perpetration.

Finally, when we separated out the different forms of IPV, it was only the impact of SS/CF on emotional IPV, which was mediated by depression. Other research has found that depression is associated with poor communication [41,42], and it may therefore be that reductions in depression, led to improved communication and therefore reduced perpetration of emotional IPV.

We acknowledge that there are some limitations. First, screening instruments were used to measure depression, rather than clinical diagnosis, although it is unclear how this would have affected our results. Second, while self-reporting reduces social desirability, it could have existed in the intervention arm. However, this seems improbable given that several measures did not differ significantly between groups and desired changes were not consistently observed across all outcomes (as reported in the main outcome study) [17]. Because our study was restricted to informal settlements, there is limited generalisability of these findings to other contexts, although this does not necessarily disprove the validity of the associations found in the data. Further research should assess the role of other forms of mental disorders prevalent in informal settlements, such as post-traumatic stress and anxiety, and alcohol and substance abuse [5,43], in mediating the relationship between SS/CF and IPV perpetration.

## Conclusion

This study examined whether improved mental health (depression) mediated the association between the SS/CF intervention and reduced IPV. The study found that the gender transformative and economic empowerment SS/CF intervention’s impact on IPV perpetration was mediated through improved depressive symptoms, suggesting mental health may be one mechanism by which the intervention was effective. Future IPV prevention interventions may consider including components that focus directly on improving depression to strengthen outcomes, particularly in disadvantaged settings.

## Paper context

Stepping Stones and Creating Futures, gender-transformative economic empowerment interventions, reduced intimate partner violence (IPV) perpetration by young men. The study highlights the link between depression and IPV across cultural and global contexts and provides new evidence that men’s reduction of IPV perpetration was attributed to improved mental health. Findings from this study can be utilized to refine intervention and address unacceptably high levels of IPV in South Africa and globally, specifically in low-income nations.
